# Supplementary material for: Two-electron transfer stabilized by excited-state aromatization
Source: Nat Commun. 2019 Nov 1;10:4983. doi: 10.1038/s41467-019-12986-w (PMC6825201; doi:10.1038/s41467-019-12986-w)
Supplement: Supplementary file 2 — Supplementary Information [file 41467_2019_12986_MOESM2_ESM.pdf]

Supplementary Information

Two-Electron Transfer Stabilized by Excited-State Aromatization

Kim et al.

Supplementary Methods

Sample Preparation. The details in synthesis and characterization of **TMTQ** are described elsewhere.[1]

Steady-state absorption spectroscopy. The sample solutions were prepared in approximately micromolar concentrations in toluene, CH_2Cl_2 and CH_3NO_2, which were purchased from Sigma-Aldrich. The steady-state absorption spectra were obtained with an UV-VIS-NIR spectrometer (Varian, Cary 5000).

Infrared (IR) spectroscopy. To obtain IR spectra, samples were prepared under the solution and solid, the KBr pallet, conditions. The IR spectra were recorded using a Bruker Vertex 70 FT-IR spectrometer. IR spectro-electrochemical data were obtained by using an optically transparent thin layer electrochemical (OTTLE) cell positioned in the sample compartment of a FT-IR Nicolet iS5 spectrometer. The working electrode (Pt minigrid, 32 wires/cm) potential was controlled with an Electrochemical Analyzer BAS 100B and referenced to the Fc/Fc^+ couple. The supporting electrolyte was a 0.1 mol/L dichloromethane solution of tetrabutylammonium hexafluorophosphate (TBA-PF_6). In OTTLE cell, optical path and sample volume is less than 0.2 mm and 0.2 mL, respectively.

Femtosecond Transient Absorption Measurements. The femtosecond pump-probe spectrometer consists of two independently-tunable home-made nonlinear optical parametric amplifiers (OPAs) pumped by a regeneratively amplified Ti:sapphire laser system (Spitfire, Spectra-Physics) and an optical detection system. The OPA is based on a non-collinearly phase-matching geometry, which was easily

color-tuned by controlling the optical delay between white light continuum seed pulses (450-1400 nm) and the visible pump pulse (400 nm) produced by using a sapphire window and a barium borate (BBO) crystal, respectively. The generated visible OPA pulses had a pulse width of ~30 fs and an average power of 5 mW in the range 500-700 nm after fused-silica prism compressor. Two OPA pulses were used as the pump and probe pulses, respectively, for pump-probe measurement.[2]

The femtosecond transient absorption spectra were also measured with pump-probe spectrometer, which is consisted of Optical Parametric Amplifiers (Palitra, Quantronix) pumped by a Ti:sapphire regenerative amplifier system (Integra-C, Quantronix) operating at 1 kHz repetition rate and an optical detection system. The generated OPA pulses had a pulse width of ~ 100 fs and an average power of 100 mW in the range 280-2700 nm which were used as pump pulses. White light continuum (WLC) probe pulses were generated using a sapphire window (3 mm of thickness) by focusing of small portion of the fundamental 800 nm pulses which was picked off by a quartz plate before entering to the OPA.[3]

After the measurements, we carefully checked absorption spectra of all compounds to detect if there were artifacts due to degradation and photo-oxidation of samples. HPLC grade solvents were used in all measurements. The three-dimensional data sets of ΔA versus time and wavelength were subjected to singular value decomposition and global fitting to obtain the kinetic time constants and their associated spectra using Surface Xplorer software (Ultrafast Systems).

The femtosecond time-resolved IR spectroscopy. For measurement of femtosecond time-resolved IR spectra, a visible pump pulse and a mid-IR probe pulse were generated by two home-built OPAs, pumped by a Ti:sapphire amplified pulse. One OPA generated a pump pulse at 570 nm with 0.4 μJ of energy by frequency doubling of its signal pulse and the other OPA generated a tunable mid-IR

probe pulse by difference frequency mixing of its signal and idler pulses. The probe pulse was sent through the sample for the time-resolved mid-IR absorbance after the optically delayed pump pulse irradiated the sample. The broadband-transmitted probe pulse was detected by a 64-element N_2(l)-cooled HgCdTe array detector that was mounted on the focal plane of a 320 mm IR monochromator with a 150 *l*/mm grating. The spectral resolution of this configuration resulted in about 1.2 cm^{-1}/pixel at 1630 cm^{-1}. Spectra spanning about 400 cm^{-1} were superposition of six 64-point spectra that overlapped by several elements. The instrument response function was typically 160 fs. The time-resolved IR spectra were obtained under flowing condition.[4,5]

Computational details. Quantum mechanical calculations for molecular geometry optimization and vibrational frequency analysis were performed by the Gaussian09 Revision E.01 program suite.[6] All calculations were carried out by the density functional theory (DFT) method with the Becke's three-parameter hybrid exchange functional and the Lee-Yang-Parr correlation functional (B3LYP) with Grimme's D3 model (EmpiricalDispersion=GD3BJ),[7,8,9] employing a basis-set of 6-311G(d,p) for all atoms.[10] The excited singlet state calculations were carried out based on time-dependent DFT (TDDFT) method with the same functional and basis-set. For the triplet state analyses, unrestricted DFT calculations were conducted with triplet spin multiplicity.

The values for dihedral angle standard deviation of [10]annulene in **TMTQ** are standard deviation of angles defined by an angle estimated between the two outer bonds among three consecutive carbon-carbon bonds.

Quantum mechanical calculations for in-depth analyses of electronic structures have been done with the Q-Chem program.[11] Excited state transition energies and optimized geometries have been computed at the time-dependent DFT (TDDFT)

level with the Tamm-Dancoff approximation (TDA),[12] the B3LYP functional and the 6-31G** basis set. Solvent effects of toluene, CH_2Cl_2 and CH_3NO_2 have been taken into account with the conductor-like polarized continuum model (C-PCM).[13,14,15]

Computation of the diabatic states was performed for the two lowest singlet excited states (S_1 and dark) by means of the Edmiston-Ruedenberg localization scheme.[16]

The biradical and multiexcitonic character of low-lying states have been computed at the restricted active space CI spin-flip method (RASCI-SF)[17] with short-range DFT exchange and correlation contributions (RASCI-srDFT).[18] The reference configuration corresponds to the lowest quintet ROHF state, RAS2 contain 4 electrons in 4 orbitals while RAS1 (RAS3) correspond to the entire set of doubly occupied (virtual) set of orbitals. Short-range version of the exchange and correlation PBE functionals was used with the range separation parameter μ = 0.4 a.u. The diradical nature of excited singlets has been characterized by means of fractional occupancy density (FOD) computed at RASCI-2SF.

Supplementary Table 1. The steady-state and FT-IR spectra and TA decay profiles of **TMTQ**.

**Solvent**	**Abs**	**FT-IR**	**TA fitting constants**			
	λ_{max} (nm)	$\lambda_{(C\equiv N)}$ (cm^{-1})	τ_1	τ_2	τ_3	τ_4
Toluene	681	2214	< 40 fs	1.3 ps	40 ps	–
CH_2Cl_2	681	2214	< 40 fs	1.0 ps	9 ps	15 ps
CH_3NO_2	681	2213	< 40 fs	0.8 ps	2.5 ps	19 ps

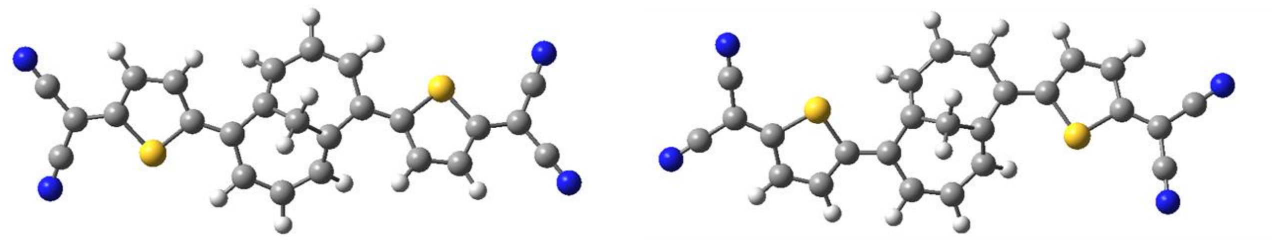

exo endo

Supplementary Figure 1. The optimized structure of **TMTQ** in the two molecular structures obtained with B3LYP-D3/6-311G(d,p). The exc conformation is 0.061 eV (1.41 kcal/mol) more stable than the endo conformation.

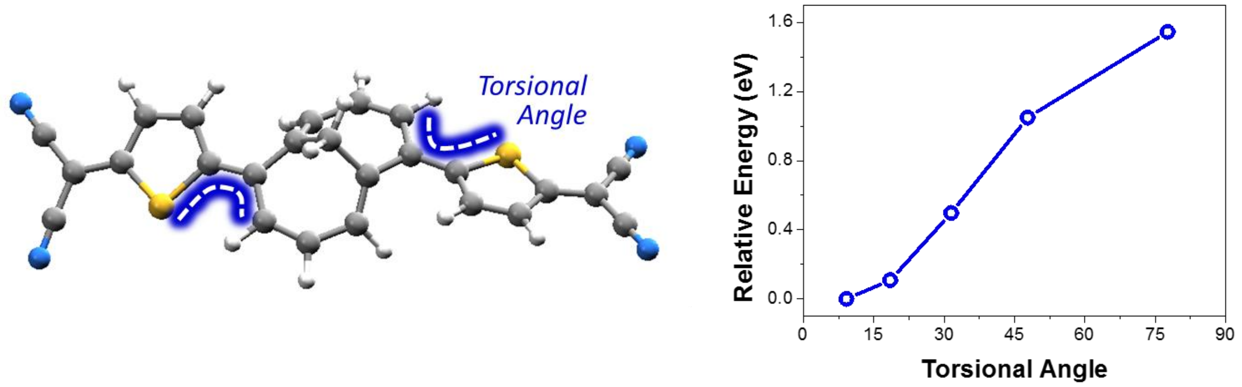

Supplementary Figure 2. The relative energy plot of **TMTQ** upon the change of torsional angle obtained by B3LYP/6-31G(d). The energy for the optimized S_0-state structure with a torsional angle of 9.16˚ was set as zero. The estimated rotational barrier of dicyanomethyl thiophene unit is more than 1 eV (24 kcal/mol), which is significantly high for a rotation of dicyanomethyl thiophene unit under room temperature condition.

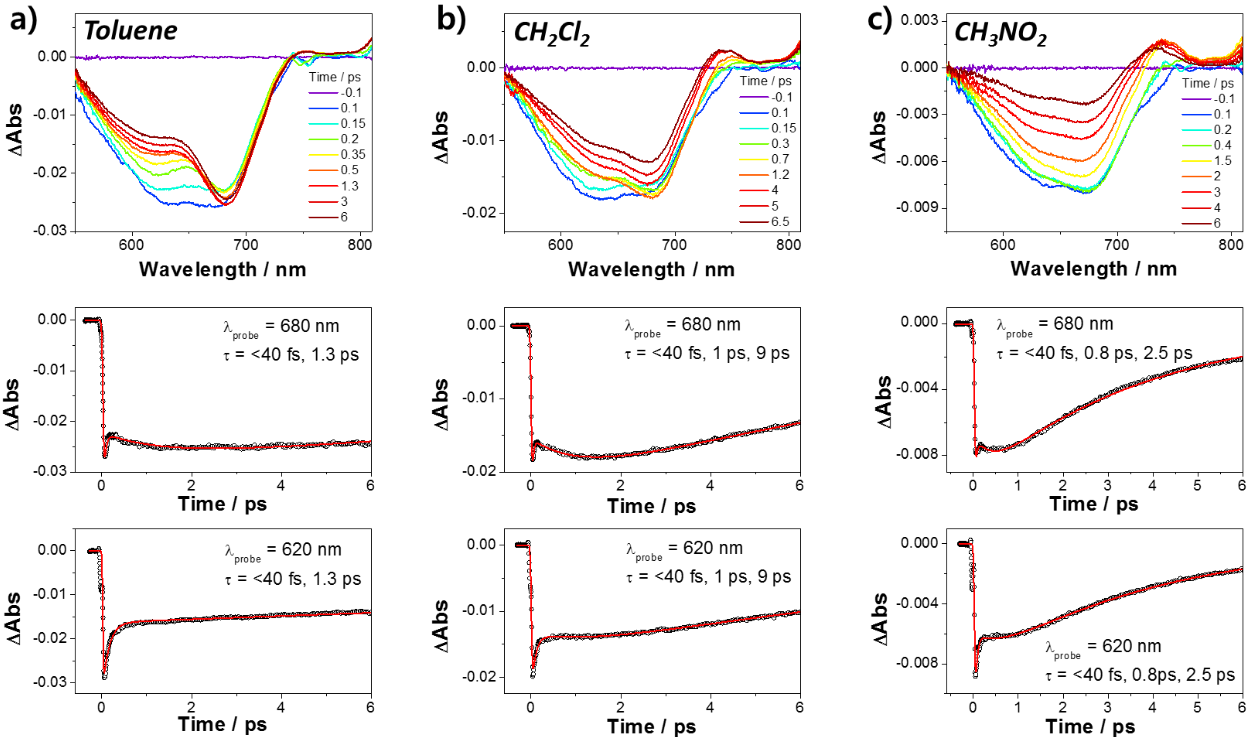

Supplementary Figure 3. TA spectra (top) and decay profiles of **TMTQ** probed at 680 nm (middle) and 620 nm (bottom) in toluene (a), CH_2Cl_2 (b), and CH_3NO_2 (c) within the first 6 ps with a broad photoexcitation centered at 580 nm.

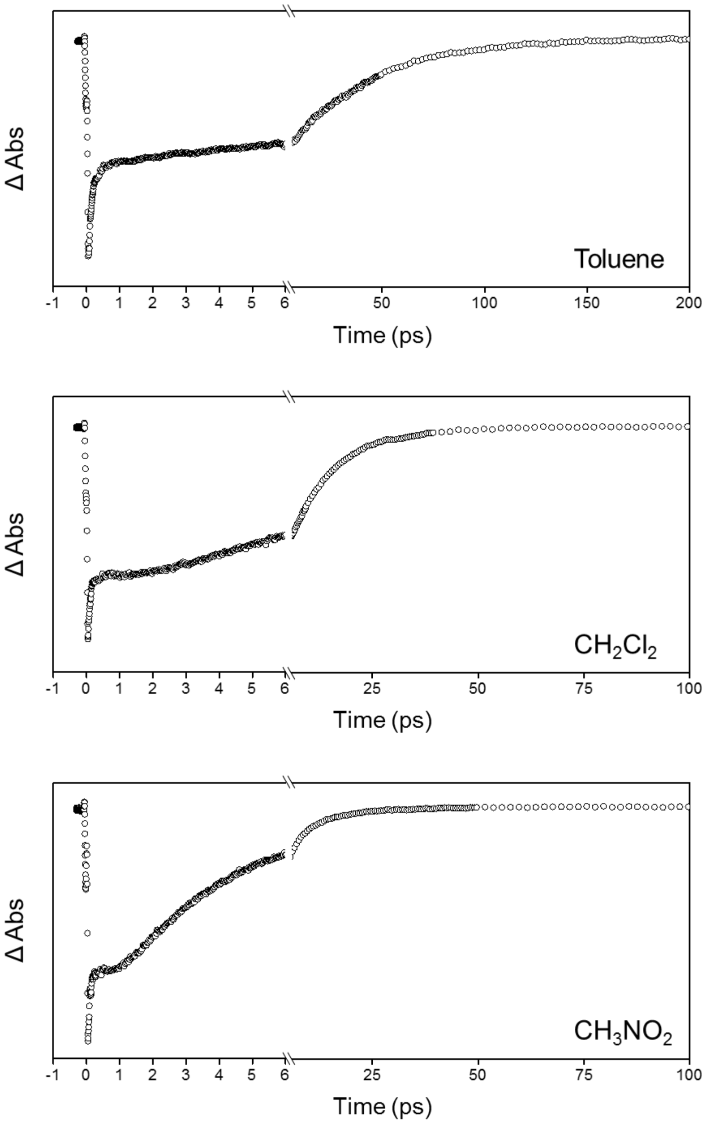

Supplementary Figure 4. TA decay profiles of **TMTQ** at 620 nm in toluene (top), CH_2Cl_2 (middle) and CH_3NO_2 (bottom) within a broad photoexcitation centered at 580 nm.

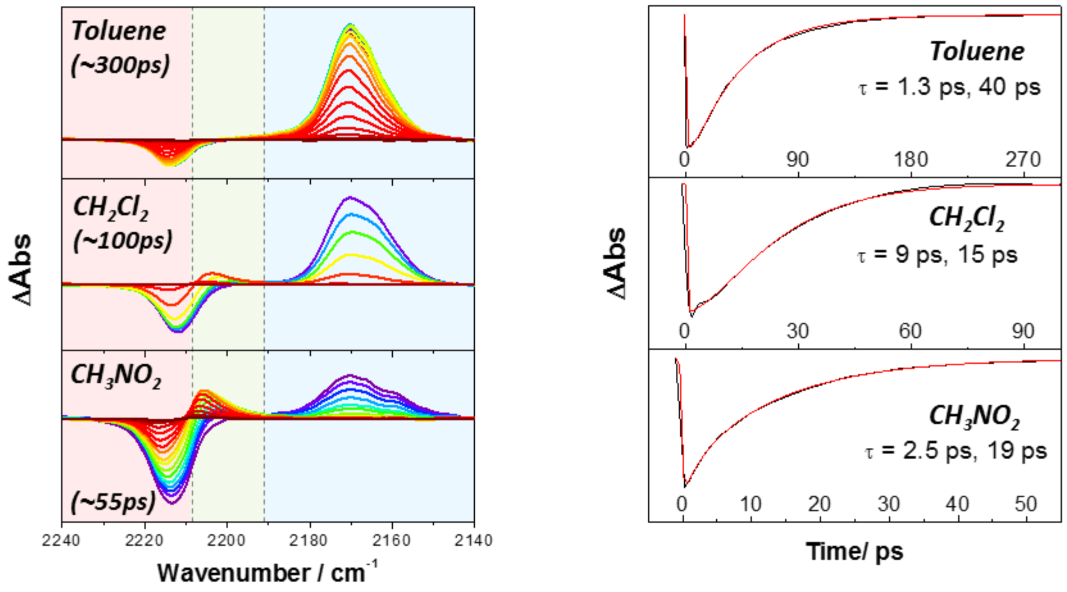

Supplementary Figure 5. The transient IR spectra (left) and decay profiles at the GSB signal (right) of **TMTQ** in toluene, CH_2Cl_2 and CH_3NO_2. In the transient IR spectra, the region for GSB signals is colored as pink. The region for the PIA band from the C≡N stretching modes in the excited state is colored as light blue. The light green region is for an additional PIA band appearing around 2205 cm^{-1} after decay of the PIA band at 2170 cm^{-1} in CH_2Cl_2 and CH_3NO_2, which is a characteristic spectral feature representing hot-vibrational bands in the ground state.

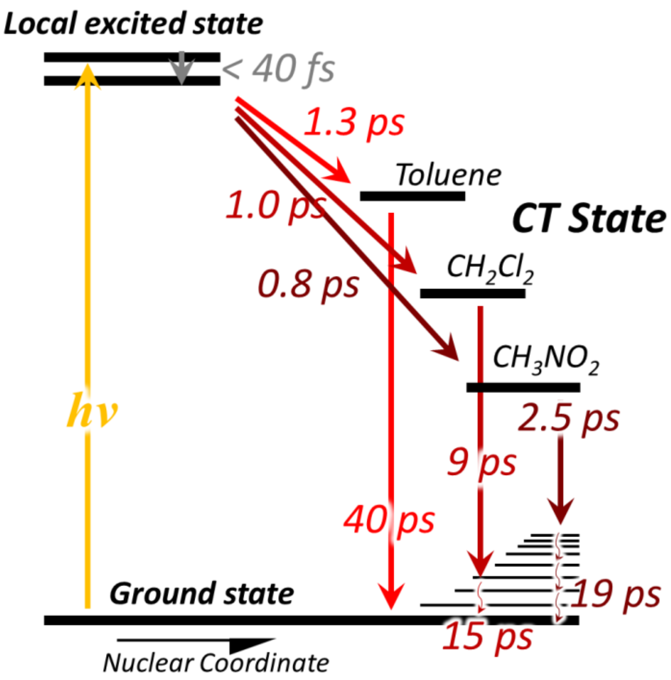

Supplementary Figure 6. The Jablonski diagram of experimentally observed excited-state dynamics of **TMTQ**.

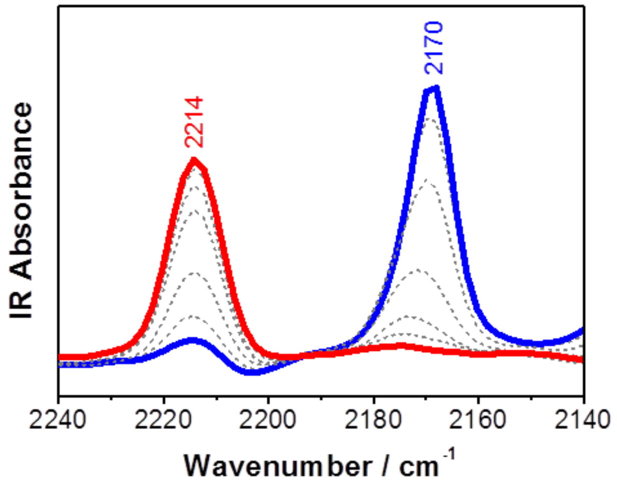

Supplementary Figure 7. The FT-IR spectra of neutral (red line) and electrochemically produced dianion (blue line) **TMTQ** in the region of 2140~2240 cm^{-1} for the C≡N stretching vibrational modes.

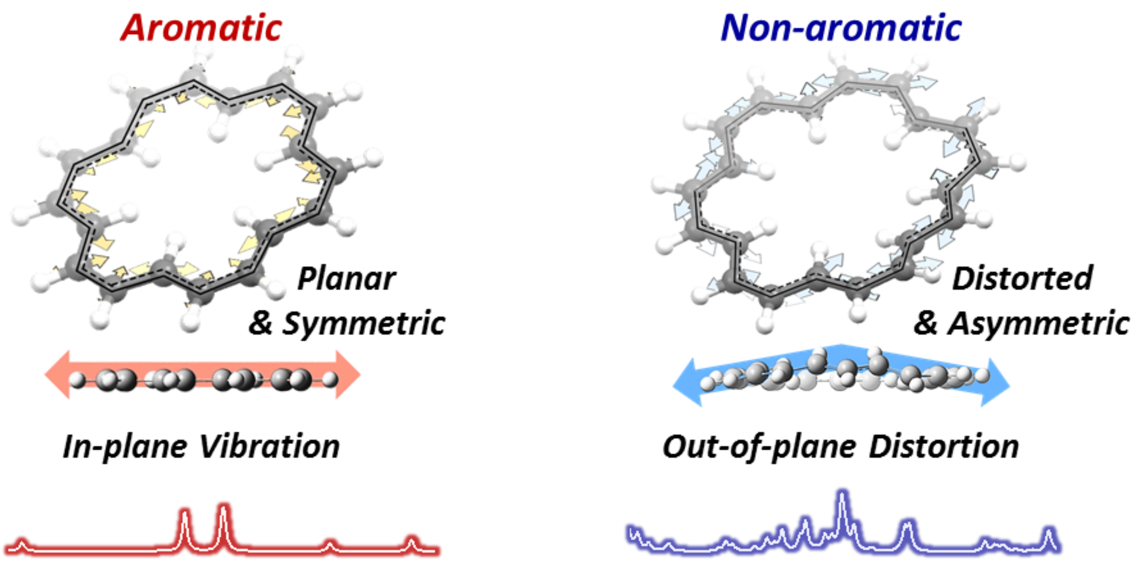

Supplementary Figure 8. Schematic illustration of IR-activity of C=C stretching vibrational bands along the molecular geometries.

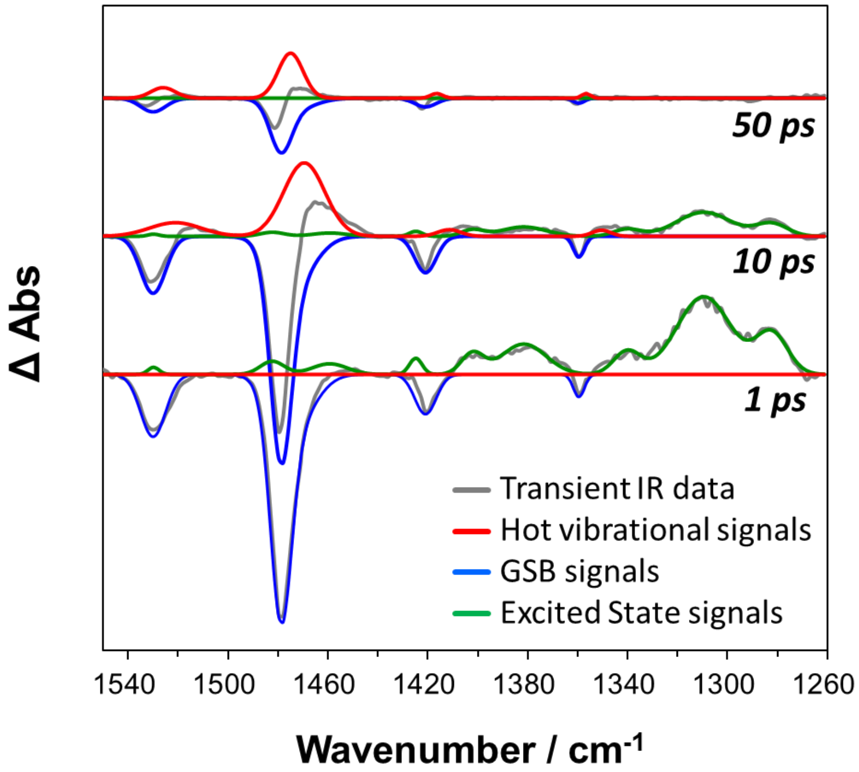

Supplementary Figure 9. Spectral analysis of transient IR spectra of **TMTQ** in CH_2Cl_2 based on the negative FT-IR spectra (blue line) and ground-state hot vibrational signals (red line).

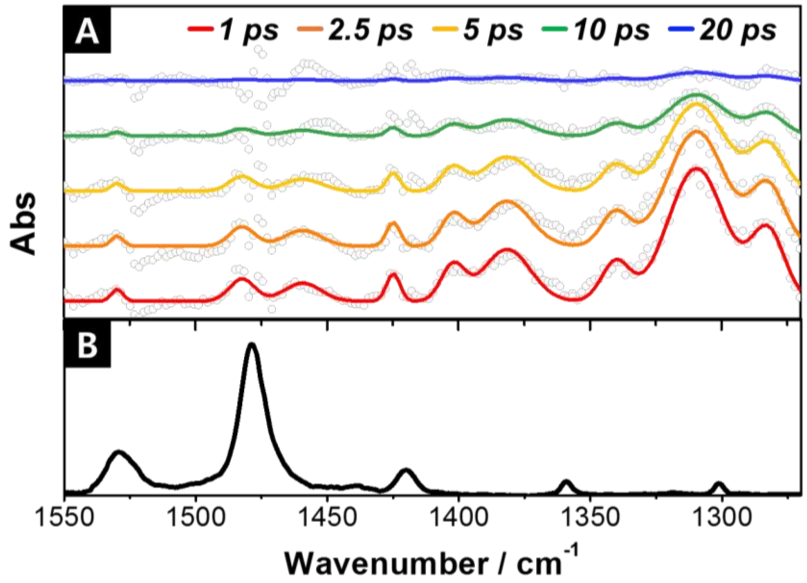

Supplementary Figure 10. The excited-state (a) and ground-state (b) IR spectra of **TMTQ**. The excited-state IR spectra were extracted from the transient IR spectra in CH_2Cl_2.

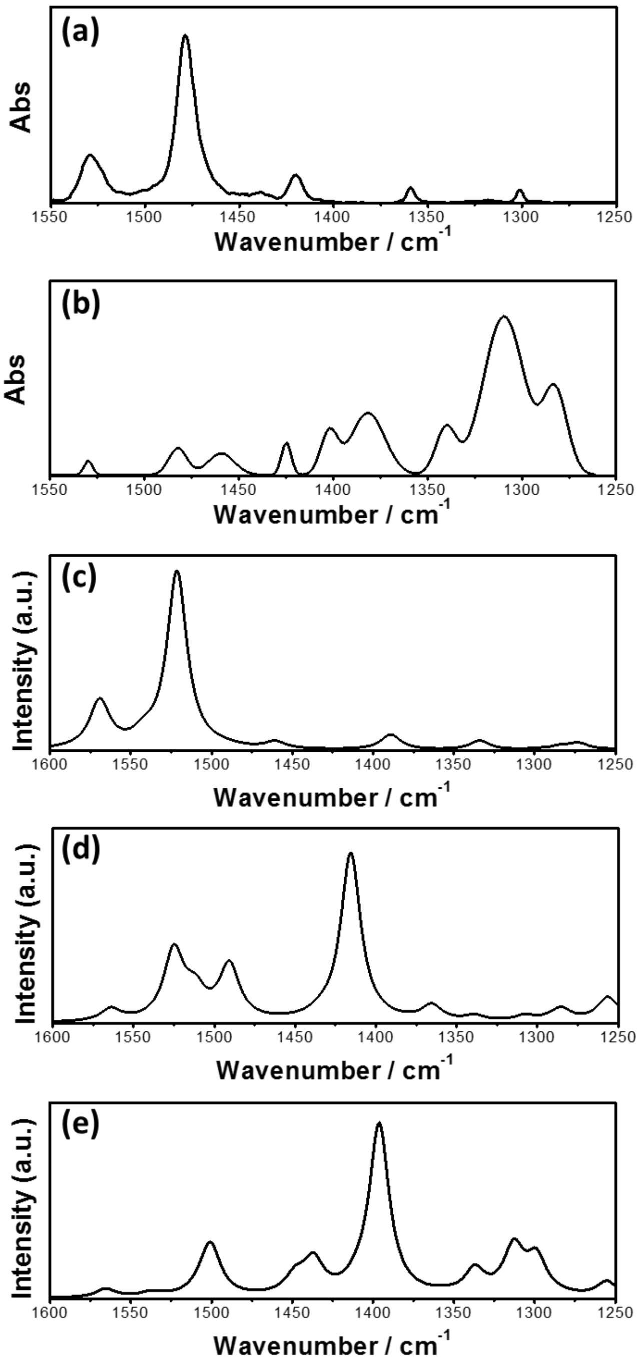

Supplementary Figure 11. The experimentally obtained (a) ground-state and (b) excited-state IR spectra of **TMTQ**. The calculated (c) S_0-state, (d) S_1-state and (e) T_1-state IR spectra of **TMTQ**.

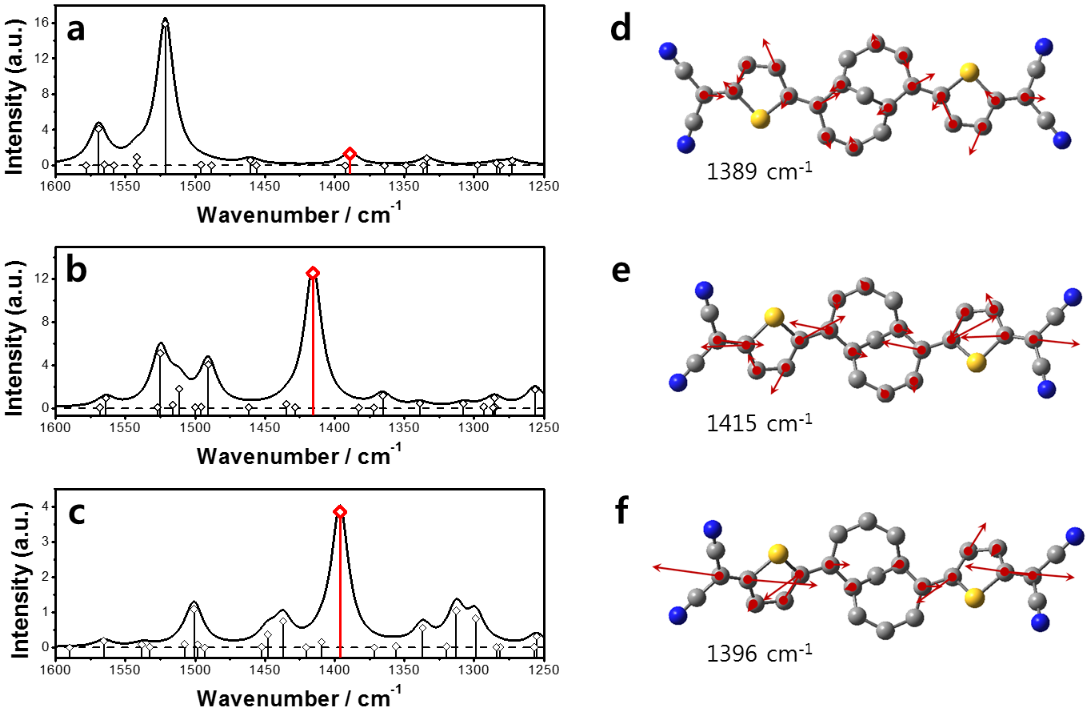

Supplementary Figure 12. The calculated IR spectra of **TMTQ** for the S_0 (a), S_1 (b) and T_1 (c) states and C=C stretching vibrational motions in the S_0 (d), S_1 (e) and T_1 (f) states (All data obtained by B3LYP-D3/6-311G(d,p)). Compared to the S_0-state and experimental IR spectra, the red-marked S_1- and T_1-state IR bands around 1400 cm^{-1}, arising from their enhanced conjugation along the long-axis in the linear geometry of **TMTQ** in the S_1 and T_1 states, are overestimated.

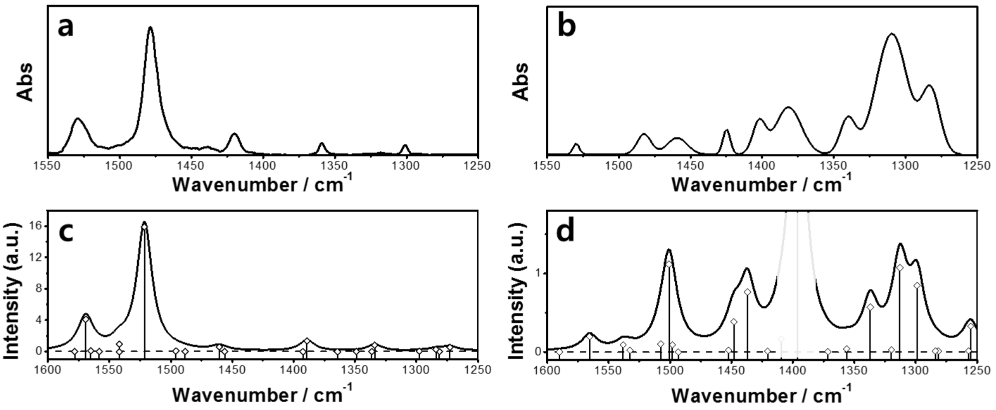

Supplementary Figure 13. The experimental ground-state (a) and excited-state (b) IR spectra and calculated S_0-state (c) and T_1-state (d) of **TMTQ** in the region for C=C stretching modes. DFT frequency calculations were carried out with B3LYP-D3/6-311G(d,p). In the IR spectra for the T_1 state (d), the intense IR band around 1400 cm^{-1} was blurred for a comparatively analysis. As explained in Figure S8, the IR band around 1400 cm^{-1} is largely overestimated. Thus, if these overestimated IR band is ignored, the T_0-state IR spectra (d) shows substantially reasonable consistency with the experimentally obtained excited-state IR spectra (b).

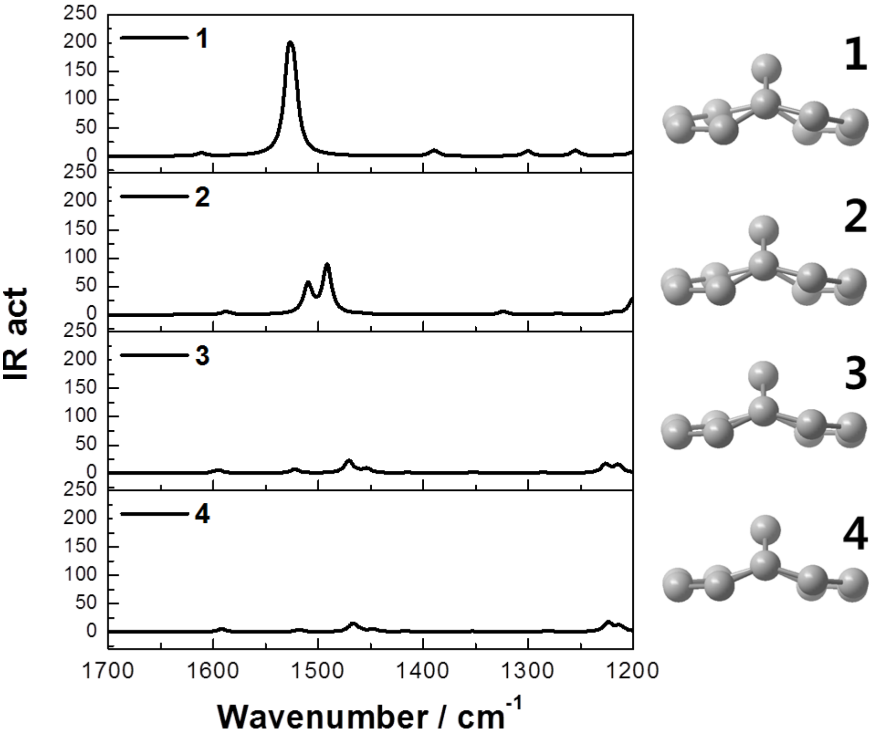

Supplementary Figure 14. The calculated IR spectra of dicationic core annulene in the T_1 state. The structures **1** and **4** of dicationic core annulene were obtained from the S_0-state and T_1-state optimized structures of **TMTQ**, respectively. **2** and **3** are intermediate structures of conformational change in going from **1** to **4**. The triplet dicationic character well reflects the quinoidal nature of **TMTQ** system with its aromaticity change.

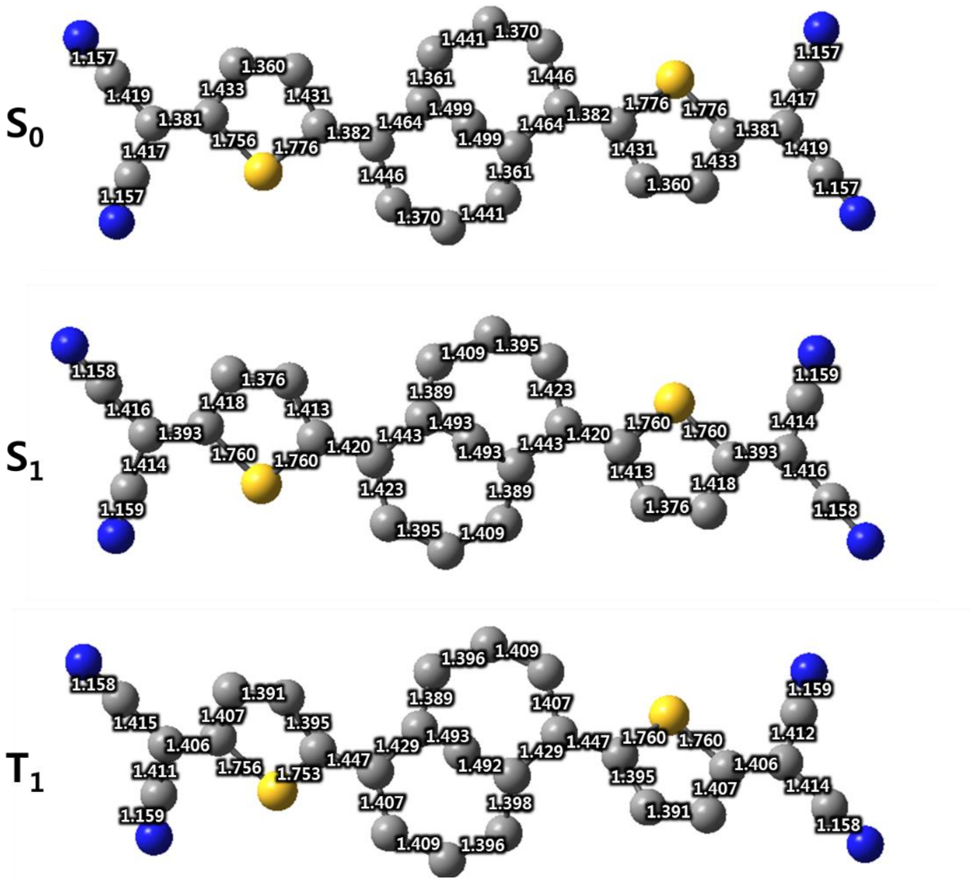

Supplementary Figure 15. The bond lengthes of **TMTQ** in S_0, S_1 and T_1 state obtained by B3LYP-D3/6-311G(d,p).

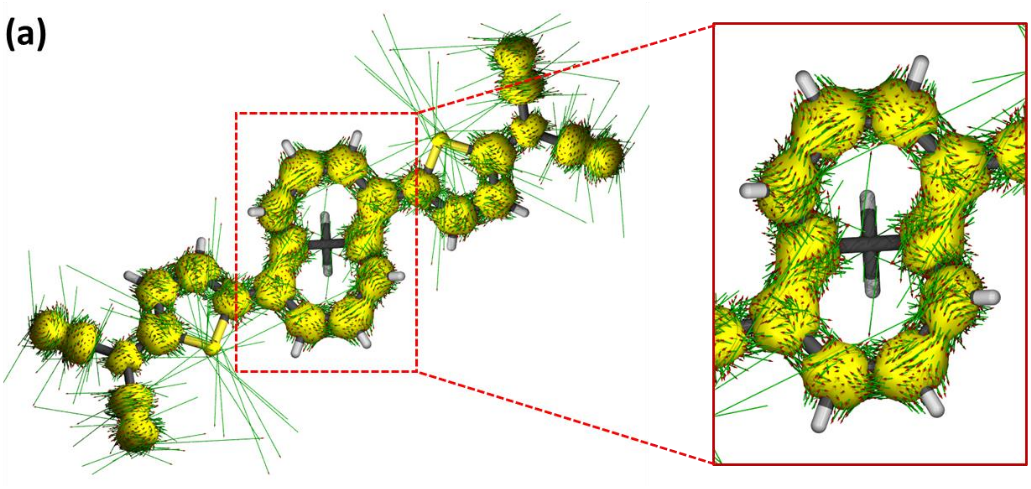

(b)

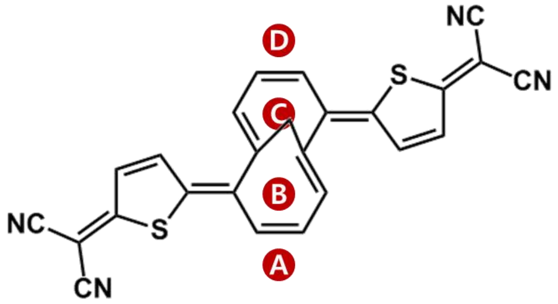

Position	NICS (ppm)
A	-1.39
B	-3.29
C	-3.29
D	-1.39

Supplementary Figure 16. (a) Anisotropy of the induced current density (ACID) plot with an isosurface value of 0.07 and (b) nucleus-independent chemical shift (NICS) values of **TMTQ** in the S_0 state. ACID plots visualize the density of electron with yellow isosurfaces and magnetically induced ring current with green arrows.[19,20] The NICS method calculate a chemical shift by magnetic shielding effect at a specific point.[21]

Supplementary Table 2. Vertical transition energies (in eV) to the two lowest singlet excited states at the ground state geometry computed at the TDDFT/TDA level with the B3LYP functional, the 6-31G** and 6-311+G** basis sets in toluene and CH_3NO_2 solution. Oscillator strength are indicated in parenthesis.

	6-31G**		6-311+G**	
	toluene	CH_3NO_2	toluene	CH_3NO_2
S_1	1.83 (3.217)	1.87 (3.185)	1.78 (3.117)	1.82 (3.081)
dark	2.13 (0.000)	2.16 (0.000)	2.12 (0.000)	2.16 (0.000)

Supplementary Table 3. Vertical transition energies (in eV) to the two lowest singlet excited states at the ground state geometry computed at the TDDFT/TDA level with different functionals and the 6-311+G** basis sets in CH_3NO_2 solution. Oscillator strength are indicated in parenthesis.

	B3LYP	CAM-B3LYP
S_1	1.82 (3.081)	1.90 (2.568)
dark	2.16 (0.000)	2.92 (0.002)

Supplementary Table 4. Vertical transition energies (in eV) to the two lowest singlet excited states at the ground state geometry computed at the RAS-2SF-*sr*PBE/6-31G(d), def2-SVP and def2-TZVP levels in vacuum. Oscillator strength are indicated in parenthesis.

	6-31G(d)	def2-SVP	def2-TZVP
dark	2.09 (0.000)	2.06 (0.000)	2.08 (0.000)
S_1	2.33 (2.953)	2.31 (2.930)	2.33 (2.945)

The results show virtually no basis-set dependence for the two lowest-lying excited singlet states. It is important to notice that multiconfigurational calculations indicate that the electronic structure of the dark state holds important multiexcitonic character (double excitation $HOMO^2 \rightarrow LUMO^2$). This might explain the different energy ordering of between dark and bright excited singlets at the Franck-Condom geometry. On the other hand, RAS-2SF-srPBE calculations have been performed in vacuum, and hence they cannot account for the solvent dependence relaxation effects on the excited state manifold.

Supplementary Table 5. Vertical deexcitation energies (in eV) from the two lowest singlet excited states to the ground state at the S_1 and dark optimized geometries respectively, computed at the TDDFT/TDA level with the B3LYP functional, the 6-31G** basis sets in toluene, CH_2Cl_2 and CH_3NO_2 solution. Oscillator strength are indicated in parenthesis.

	Toluene	CH_2Cl_2	CH3NO2
S_1	1.73 (3.523)	1.41 (3.295)	1.31 (30161)
dark	1.94 (0.001)	1.97 (0.003)	1.98 (0.005)

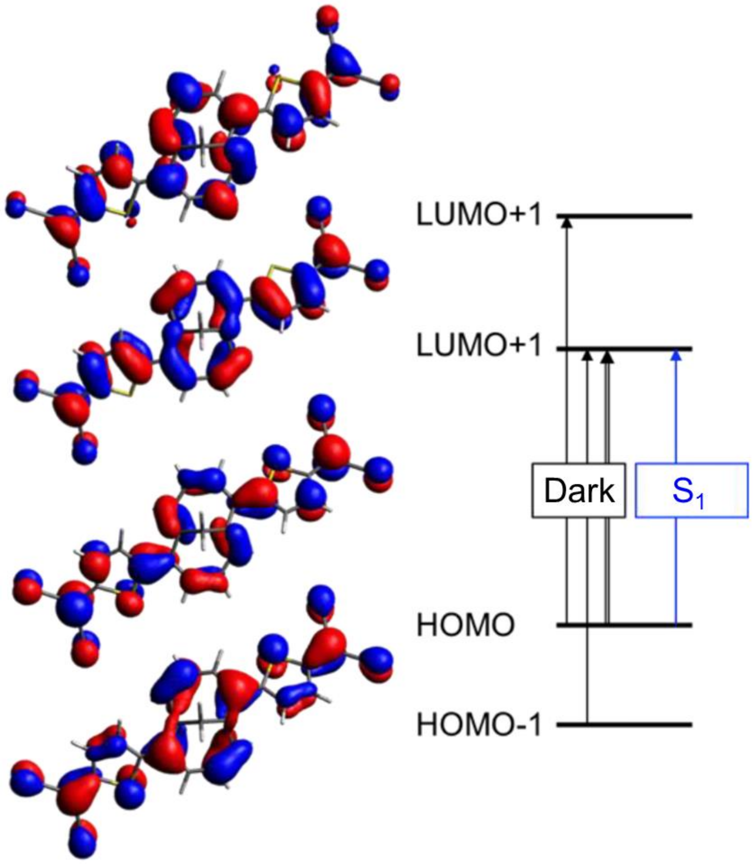

Supplementary Figure 17. Molecular orbitals mainly participating in the transition to the dark and S_1 states of **TMTQ**. Double arrow indicates multiexcitonic character.

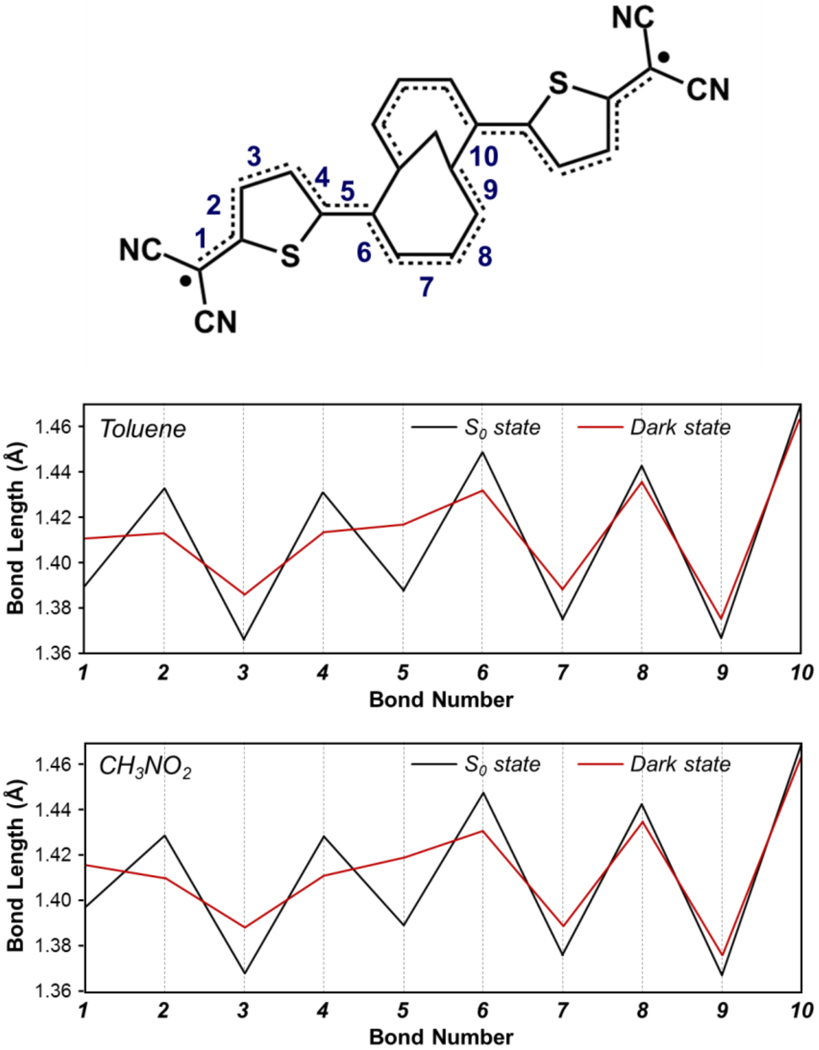

Supplementary Figure 18. The carbon-carbon bond length distribution plot of **TMTQ** in the S_0 and dark states. The molecular structure is drawn based on the bond length distribution in the dark state.

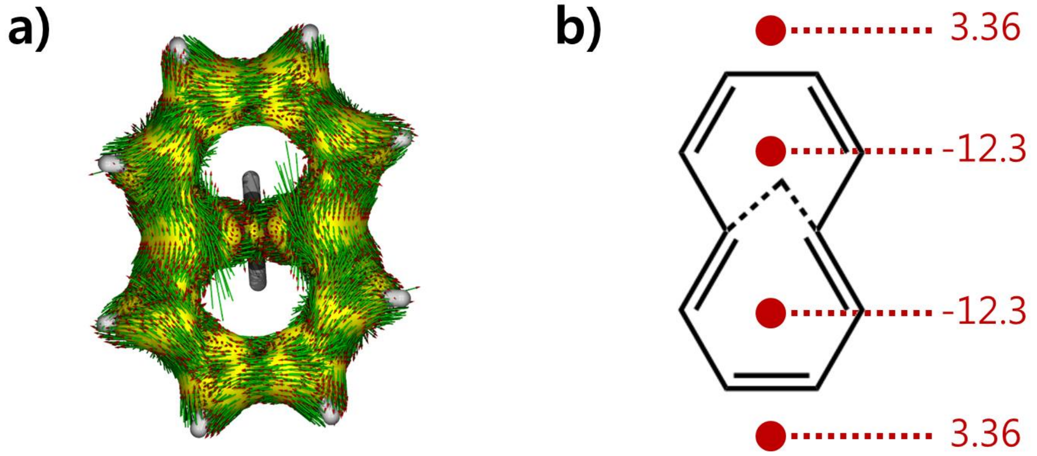

Supplementary Figure 19. (a) ACID plot with an isosurface value of 0.07 and (b) NICS values of triplet planar M10A dication. The molecular structure was obtained based on the T_1-state optimized structure of **TMTQ**. Compared to the ACID and NICS results for M10A of **TMTQ** in the S_0 state, the clorkwise ring-current with continuous isosurface in the ACID plot and large negative NICS values at inner annulene represent the aromatic nature of core 8π annulene. In both structure, the methylene bridge ($-CH_2-$) faces below the plane.

Supplementary References

[1] Streifel, B. C., Zafra, J. L., Espejo, Z. G., Gómez-García, C. J., Casado, J., and Tovar, J. D. Unusually Small Singlet-Triplet Gap in a Quinoidal 1,6-Methano[10]annulene Resulting from Baird's 4n π-Electron Triplet Stabilization. *Angew. Chem. Int. Ed.* **2015**, *54*, 5888–5893.

[2] Zhang, Y.; Oh, J.; Wang, K.; Chen, C.; Caso, W.; Park, K. H.; Kim, D.; Jiang, J. Heteroleptic Tetrapyrrole-Fused Dimeric and Trimeric Skeletons with Unusual Non-Frustrated Fluorescence. *Chem. Eur. J.* **2016**, *22*, 4492–4499.

[3] Sung, Y. M.; Yoon, M. C.; Lim, J. M.; Rath, H.; Naoda, K.; Osuka, A.; Kim, D. Reversal of Hückel (anti)aromaticity in the lowest triplet states of hexaphyrins and spectroscopic evidence for Baird's rule. *Nat. Chem.* **2015**, *7*, 418-422.

[4] Kim, S.; Lim, M. Protein Conformation-Induced Modulation of Ligand Binding Kinetics: A Femtosecond Mid-IR Study of Nitric Oxide Binding Trajectories in Myoglobin. *J. Am. Chem. Soc.* **2005**, *127*, 8908 – 8909.

[5] Kim, S.; Park, J.; Lee, T.; Lim, M. Direct Observation of Ligand Rebinding Pathways in Hemoglobin Using Femtosecond Mid-IR Spectroscopy. *J. Phys. Chem. B* **2012**, *116*, 6346 – 6355.

[6] Gaussian 09, Revision E.01, Frisch, M. J.; Trucks, G. W.; Schlegel, H. B.; Scuseria, G. E.; Robb, M. A.; Cheeseman, J. R. et al. Gaussian, Inc., Wallingford CT, **2009**.

[7] Becke, A. D. Density-functional thermochemistry. III. The role of exact exchange. *J. Chem. Phys.* **1993**, *98*, 5648–5652.

[8] Lee, C.; Yang, W.; Parr, R. G. Development of the Colle-Salvetti correlation-energy formula into a functional of the electron density. *Phys. Rev. B* **1988**, *37*, 785–789.

[9] Stephens, P. J.; Devlin, F. J.; Chabalowski, C. F.; Frisch, M. J. Ab Initio Calculation of Vibrational Absorption and Circular Dichroism Spectra Using

Density Functional Force Fields. *J. Phys. Chem.* **1994**, *98*, 11623–11627.

[10] Krishnan, R.; Binkley, J. S.; Seeger, R.; Pople, J. A. Self-consistent molecular orbital methods. XX. A basis set for correlated wave functions. *J. Chem. Phys.* **1980**, *72*, 650-654.

[11] Shao, Y.; Gan, Z.; Epifoanovsky, E.; Gilber, A. T. B.; Wormit, M. et al. Advances in molecular quantum chemistry contained in the Q-Chem 4 program package. *Mol. Phys.* **2015**, *113*, 184–215.

[12] Hirata, S.; Head-Gordon, M. Time-dependent density functional theory within the Tamm-Dancoff approximation. *Chem. Phys. Lett.* **1999**, *314*, 291–299.

[13] Truong, T. N.; Stefanovich, E. V. A new method for incorporating solvent effect into the classical, ab initio molecular orbital and density functional theory frameworks for arbitrary shape cavity. *Chem. Phys. Lett.* **1995**, *240*, 253–260.

[14] Barone, V.; Cossi, M. Quantum calculation of molecular energies and energy gradients in solution by a conductor solvent model. *J. Phys. Chem. A* **1998**, *102*, 1995–2001.

[15] Cossi, M.; Rega, N.; Scalmani, G.; Barone, V. Energies, structures, and electronic properties of molecules in solution with the C-PCM solvation model. *J. Comput. Chem.* **2003**, *24*, 669–681.

[16] Subotnik, J. E.; Cave, R. J.; Steele, R. P.; Shenvi, N. The initial and final states of electron and energy transfer processes: Diabatization as motivated by system-solvent interactions. *J. Chem. Phys.* **2009**, *130*, 234102.

[17] Casanova, D.; Head-Gordon, M. Restricted active space spin-flip configuration interaction approach: theory, implementation and examples. *Phys. Chem. Chem. Phys.* **2009**, *11*, 9779–9790.

[18] Casanova, D. Short-range density functional correlation within the restricted

active space CI method. *J. Chem. Phys.* **2018**, *148*, 124118.

[19] Herges, R.; Geuenich, D. Delocalization of electrons in molecules. *J. Phys. Chem. A* **2001**, *105*, 3214 –3220.

[20] Geuenich, D.; Hess, K.; Kçhler, F.; Herges, R. Anisotropy of the induced current density (ACID), a general method to quantify and visualize electronic delocalization. *Chem. Rev.* **2005**, *105*, 3758–3772.

[21] Schleyer, P. v. R.; Maerker, C.; Dransfeld, A.; Jiao, H.; Hommes, N. J. R. v. E. Nucleus-independent chemical shifts: a simple and efficient aromaticity probe. *J. Am. Chem. Soc.* **1996**, *118*, 6317–6318.
