# Supplementary material for: Two-electron transfer stabilized by excited-state aromatization
Source: Nat Commun. 2019 Nov 1;10:4983. doi: 10.1038/s41467-019-12986-w (PMC6825201; doi:10.1038/s41467-019-12986-w)
Supplement: Supplementary file 3 — Description of Additional Supplementary Files [file 41467_2019_12986_MOESM3_ESM.pdf]

Description of Additional Supplementary Files

File Name: Supplementary Data 1

Description: Optimized structure of TMTQ for the S0 state with B3LYP(GD3BJ)/6-311G(d,p)

File Name: Supplementary Data 2

Description: Optimized structure of TMTQ for the S1 state with B3LYP(GD3BJ)/6-311G(d,p)

File Name: Supplementary Data 3

Description: Optimized structure of TMTQ for the T1 state with B3LYP(GD3BJ)/6-311G(d,p)
