# Supplementary material for: Two-electron transfer stabilized by excited-state aromatization
Source: Nat Commun. 2019 Nov 1;10:4983. doi: 10.1038/s41467-019-12986-w (PMC6825201; doi:10.1038/s41467-019-12986-w)
Supplement: Supplementary file 4 — Supplementary Data 1 [file 41467_2019_12986_MOESM4_ESM.pdf]

Supplementary Data1

Two-Electron Transfer Stabilized by Excited-State Aromatization

Supplementary Data1

Supplementary Data 1. Optimized structure of **TMTQ** for the S_0 state with B3LYP(GD3BJ)/6-311G(d,p)

S	-4.70703600	-1.05232400	-0.33724400
S	4.70703400	1.05234400	-0.33719900
N	-8.05879900	-2.19687200	-1.41032400
N	-8.87238700	2.00790400	-0.12247300
N	8.87240200	-2.00787100	-0.12256100
N	8.05878300	2.19695200	-1.41025000
C	-2.11628700	-0.43115200	0.46726100
C	-1.73638500	-1.81272200	0.27471600
C	-0.48476500	-2.33251100	0.07244100
C	0.78302400	-1.64793400	0.06308800
C	0.48476600	2.33250500	0.07258400
C	1.73638700	1.81270400	0.27482200
C	2.11628900	0.43112100	0.46727900
C	-0.78302300	1.64792900	0.06319400
C	-3.42556500	-0.01445000	0.32229900
C	-3.97351100	1.25907000	0.67756000
C	-5.30705300	1.38822900	0.44134900
C	-5.90591800	0.21345900	-0.12042300
C	3.42556600	0.01442900	0.32228500
C	3.97351400	-1.25911300	0.67746400
C	5.30705500	-1.38825700	0.44123800
C	5.90591700	-0.21345300	-0.12046400
C	-7.23497300	0.04828100	-0.45787300
C	7.23497000	-0.04825400	-0.45791300
C	8.15129300	-1.11735900	-0.28046900
C	7.71053600	1.17936000	-0.98376100
C	-7.71054200	-1.17930100	-0.98379700

C	-8.15129600	1.11737400	-0.28035500
C	1.04249300	-0.51613000	0.77218000
C	-1.04248900	0.51608100	0.77221600
C	0.00000300	-0.00005400	1.71844200
H	-2.55924700	-2.51434100	0.18249100
H	-0.45590900	-3.37727200	-0.22320600
H	1.53155800	-2.02833600	-0.62398100
H	0.45590800	3.37728500	-0.22299600
H	2.55924800	2.51432900	0.18263900
H	-1.53156000	2.02837300	-0.62384900
H	-3.36105500	2.02541900	1.13050700
H	-5.89692300	2.26547700	0.66726100
H	3.36106000	-2.02549000	1.13036600
H	5.89692600	-2.26551900	0.66709200
H	-0.39815900	-0.79700200	2.34591000
H	0.39816900	0.79685500	2.34595800
