# Supplementary material for: Two-electron transfer stabilized by excited-state aromatization
Source: Nat Commun. 2019 Nov 1;10:4983. doi: 10.1038/s41467-019-12986-w (PMC6825201; doi:10.1038/s41467-019-12986-w)
Supplement: Supplementary file 5 — Supplementary Data 2 [file 41467_2019_12986_MOESM5_ESM.pdf]

Supplementary Data2

Two-Electron Transfer Stabilized by Excited-State Aromatization

Supplementary Data2

Supplementary Data 2. Optimized structure of **TMTQ** for the S_1 state with B3LYP(GD3BJ)/6-311G(d,p)

S	4.50109300	-1.01089800	0.40846800
S	-4.50109400	1.01092200	0.40842100
N	7.61900600	-2.19792400	2.00113000
N	8.77523700	1.89798000	0.63111700
N	-8.77519800	-1.89799200	0.63134100
N	-7.61902700	2.19805200	2.00097500
C	2.05612000	-0.43397700	-0.79587300
C	1.71538800	-1.81223600	-0.69756900
C	0.45032800	-2.38173100	-0.55284200
C	-0.80416800	-1.74037100	-0.57060200
C	-0.45033000	2.38170500	-0.55296900
C	-1.71539000	1.81220200	-0.69766500
C	-2.05612200	0.43393800	-0.79589400
C	0.80416600	1.74034400	-0.57069600
C	3.36666000	0.00623300	-0.47325800
C	3.97885900	1.23193900	-0.81744000
C	5.28811700	1.35002400	-0.40822100
C	5.76495200	0.20679400	0.28336700
C	-3.36666200	-0.00625600	-0.47325300
C	-3.97886000	-1.23198100	-0.81736700
C	-5.28811700	-1.35004500	-0.40813800
C	-5.76495300	-0.20677800	0.28338700
C	7.03414900	0.01346900	0.82529200
C	-7.03414800	-0.01342400	0.82530500
C	-8.00499400	-1.03874300	0.72931500
C	-7.37727500	1.19398800	1.47601700
C	7.37727700	-1.19391000	1.47606600

C	8.00499500	1.03878200	0.72924600
C	-1.02742900	-0.50601800	-1.16892500
C	1.02742600	0.50595800	-1.16895400
C	-0.00000200	-0.00005500	-2.12837600
H	2.54724700	-2.49551300	-0.56186800
H	0.44964300	-3.42200700	-0.24263600
H	-1.60010300	-2.20313200	0.00140000
H	-0.44964500	3.42199700	-0.24282000
H	-2.54724900	2.49548600	-0.56199900
H	1.60010100	2.20313600	0.00127900
H	3.46122700	1.98329800	-1.39699000
H	5.92276300	2.20391200	-0.59951700
H	-3.46122900	-1.98337100	-1.39687600
H	-5.92276300	-2.20394400	-0.59938700
H	0.39543000	-0.80059100	-2.75121600
H	-0.39543500	0.80044600	-2.75125800
