# Supplementary material for: Two-electron transfer stabilized by excited-state aromatization
Source: Nat Commun. 2019 Nov 1;10:4983. doi: 10.1038/s41467-019-12986-w (PMC6825201; doi:10.1038/s41467-019-12986-w)
Supplement: Supplementary file 6 — Supplementary Data 3 [file 41467_2019_12986_MOESM6_ESM.pdf]

Supplementary Data3

Two-Electron Transfer Stabilized by Excited-State Aromatization

Supplementary Data

Supplementary Data 3. Optimized structure of **TMTQ** for the T_1 state with B3LYP(GD3BJ)/6-311G(d,p)

S	4.31034100	-0.96458600	0.51387100
S	-4.31033800	0.96461300	0.51383700
N	7.19248500	-2.04876800	2.59661400
N	8.71470400	1.74475700	0.80588100
N	-8.71470200	-1.74471300	0.80601000
N	-7.19247300	2.04890600	2.59653400
C	2.02312600	-0.47024400	-1.01684000
C	1.67366300	-1.83130800	-0.93946000
C	0.38931700	-2.39964900	-0.83375700
C	-0.85057200	-1.75861100	-0.86358800
C	-0.38932100	2.39960400	-0.83388200
C	-1.67366700	1.83125800	-0.93955200
C	-2.02313000	0.47019000	-1.01685800
C	0.85056800	1.75856400	-0.86368300
C	3.34611500	-0.03952800	-0.61995500
C	4.04430700	1.08737600	-1.05679200
C	5.31927400	1.20932100	-0.51501400
C	5.64698700	0.16409700	0.36760500
C	-3.34611800	0.03949400	-0.61994500
C	-4.04431200	-1.08743200	-1.05671900
C	-5.31927700	-1.20934800	-0.51493000
C	-5.64698600	-0.16407800	0.36763600
C	6.85193300	-0.00847900	1.07050700
C	-6.85192900	0.00853700	1.07053400
C	-7.88712700	-0.94554700	0.93818000
C	-7.05789700	1.12249500	1.91400600
C	7.05790500	-1.12239300	1.91403700

C	7.88713100	0.94559800	0.93809800
C	-1.03975100	-0.48757800	-1.41350900
C	1.03974500	0.48750300	-1.41353800
C	-0.00000400	-0.00006300	-2.36605200
H	2.49153100	-2.52025400	-0.75507200
H	0.38290400	-3.43686600	-0.51403100
H	-1.66907400	-2.23902200	-0.34055800
H	-0.38290700	3.43683800	-0.51421200
H	-2.49153500	2.52021400	-0.75519700
H	1.66907100	2.23900300	-0.34068100
H	3.62999500	1.77239600	-1.78299400
H	6.01644400	2.00224500	-0.74772600
H	-3.63000300	-1.77249100	-1.78288600
H	-6.01644800	-2.00228400	-0.74759700
H	0.37917300	-0.80882900	-2.98839600
H	-0.37918400	0.80867000	-2.98843700
